# Vocal fold superficial layer of lamina propria histology after the position of mucosa pediculated flap: canine experimental study

**DOI:** 10.1016/S1808-8694(15)31329-X

**Published:** 2015-10-20

**Authors:** David Greco Varela, Marcos Grellet

**Affiliations:** ^1^ Master in Otorhinolaryngology, Residence Hospital Santa Izabel; ^2^ Ph.D., Associate Professor, Department of Ophthalmology and Otorhinolaryngology and Head and Neck Surgery, Medical School, Ribeirão Preto - USP

**Keywords:** vocal fold, collagen, sulci, surgery

## Abstract

**M**any techniques were applied to treat patients with sulcus vocalis and scarred vocal folds. Their results were not good enough. In the Technique of Vocal Fold Pediculated Mucosa Flap, an anterior pediculated flap of vocal fold is positioned on the superficial layer of the lamina propria, below the free margin. **Aim**: To describe histological postoperative findings on the superficial layer of lamina propria during the application of the technique Vocal Fold Pediculated Mucosa Flap. The following parameters were compared between tested and control groups: total, type I and type III collagen and number of cellular nucleus. **Study design**: experimental. **Material and Method**: Fifteen dogs were used. One vocal fold was submitted to the intervention and the other was left as control. Each group of three dogs was sacrificed on 10, 30, 90, 180 and 360 days after the experimental surgery. Hematoxylin and eosin (H.E.) and Syrius Red were the staining techniques used. **Results**: Type I and total collagen suggested increased results in the tested group on postoperative days 90 and 180, nevertheless there was statistical significance only on postoperative day 180 (p<0.05). Type III collagen group area was less significant than the control group on postoperative day 180 (p<0.05). The number of cellular nucleus was increased on the 10th postoperative day, but decreased after the 30th day. **Discussion**: The findings about total and type I collagen and the amount of cellular nucleus on the superficial layer of lamina propria were similar to laryngeal postoperative studies in dogs. More complex studies would contribute with new data about the present subject.

## INTRODUCTION

The lamina propria has a key role in viscosity, contractility and formation of vocal fold mucosa wave. Its good functioning is determinant for the onset and maintenance of sustained vibration in Bernoulli's phenomenon^1^.

Extracellular matrix of lamina propria superficial layer comprises protein fibers and interstitial fibers^2^. In humans, protein fibers are distributed parallel to the free margin. Among them, collagen fibers are responsible for maintaining the local architecture, osmotic regulation and viscosity^3^. Type I collagen fibers are larger, better visualized and more rigid. Type III fibers are thinner, flexible and detected in initial scarring processes^4^.

Among the organic causes of incomplete glottic closure, vocal sulci and scarring lesions^5^ comprise the group of affections that require a special approach6., 7.. The composition of lamina propria suffers many pathological affections, whose solution has not been fully understood by Otorhinolaryngologists yet.

Surgical techniques using implants such as fat implants8., 9., intact or solubilized temporalis fascia implant^10^, autologous collagen11., 12. and hyaluronic acid were tested but the results obtained to present have not been universally accepted. Pontes et al.^13^ managed to reconstitute the subepithelial area using a stripping mucosa technique, which does not require the use of grafts or implants.

Gray et al., in 2003, perceived a new era in Laryngology when they developed studies with pluripotent cells for extracellular matrix recomposition, however, it is an option still in development and of high cost14., 15..

Grellet^16^ proposed a new surgical technique to approach vocal sulci. It consists of rotation of mucosa pediculated flap on the superior surface of the vocal mucosa, positioned under the surface of the detached subepithelial region, close to the free margin. It is an alternative technique that tries to improve glottic closure and reconstitution of mucosa wave.

## OBJECTIVES

To describe histological findings of lamina propria superficial layer of dogs when applying a vocal fold mucosa pediculated flap, seen on days 10, 30, 90, 180 and 360 after the surgical intervention. To check chronological affections that occurred in each period of observation in the tested groups compared to the control group, analyzing levels of the following elements: total collagen, type I and type III collagen and cell nuclei.

## MATERIAL AND METHOD

We conducted an experimental study in dogs that was evaluated and approved by the Ethics Committee on Animal Studies, Campus USP - Ribeirão Preto (Protocol 03.1.324.53.6).

We selected 15 healthy adult dogs weighing on average 12 to 17 Kg who did not have laryngeal lesions upon inspection under surgical microscope and contact laryngoscopy (Hamou I, Karl Storz 26156B). They were all animals from the Animal Laboratory, Medical School, Ribeirão Preto - USP. Surgical interventions were performed between April 2002 and May 2003. The right vocal fold was taken as the tested one and the left vocal fold was used as control in all dogs.

The studied dogs were submitted to venous general anesthesia, orotracheal intubation and ventilation by assisted pressure. Anesthetic induction was made with 50mg/kg ketamine, associated with 1ml xylazin. After intubation, anesthesia was maintained with sodium thiopental 40mg/kg. Pressure ventilation was determined by device Takaoka (K. TAKAOKA, model 850-10) with 15-sec cycles. We performed antibiotic prophylaxis with Kefazol, 30 to 40mg/kg.

**Figure 1:**
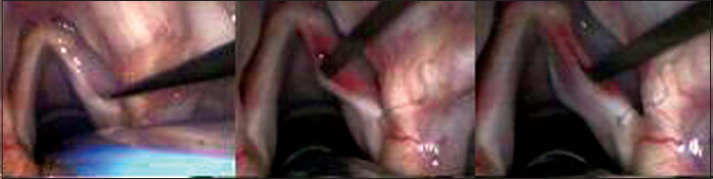
Incision made on the upper surface of the vocal fold and subepithelial detachment.

**Figure 2:**
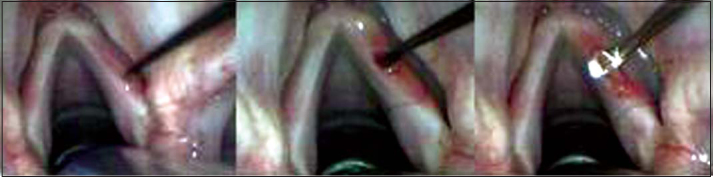
Incision on the upper surface of the vocal fold, 2 millimeters laterally to the first incision and detachment under the region delimited by two incisions.

**Figure 3:**
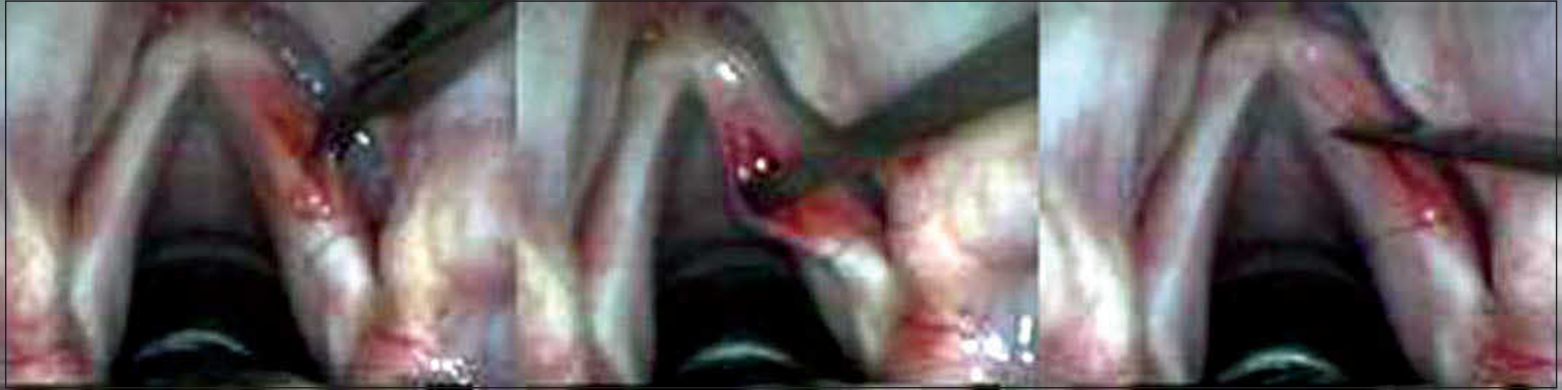
Performance of pediculated graft, positioning on subepithelial region and fixation with fibrin glue.

For the surgical intervention, we used: straight and 90º angle micro-knives, micro-scissors, curved and straight micro-detachers, and aspirators (Karl Storz, Tuttlingen, 1999). The microscope used was M-900 (DF Vasconcellos, Sao Paulo) with 400mm lenses, MC-A400. The technique consisted of making a vocal fold mucosa pediculated flap with anterior irrigation (2cm × 0.2cm) and positioning it on lamina propria superficial layer, on the region of the free margin (Figures 1, 2 and 3).

At the end of the surgery, we positioned: 1) mucosa superior surface of the pediculated flap in contact with the inferior surface of the subepithelial region, close to the free margin, and 2) inferior surface of pediculated flap in contact with the surface of the detached bed of the lamina propria.

In the first 72 hours after the surgery, we prescribed liquid diet to try to help vocal rest and scarring. Dogs were sacrificed on days 10, 30, 90, 180 and 360 after the surgery, so as to assess the behavior of the pediculated flap and scarring process of the lamina propria superficial layer at short and long term. We used an overdosage of intravenous sodium thiopental followed by 10ml of potassium chloride at 19.1% for the sacrifice. To collect the vocal folds, we made a medial cervical longitudinal incision with exposure of larynx through laryngofissure.

Vocal folds were soaked in paraffin in the longest axis direction and then transversal sections were made between the anterior and medium thirds of the extension. Staining substance Syrius Red^17^ was used to analyze total, type I and type III collagen under polarized light. Hematoxylin and Eosin aimed at identifying the cell nuclei.

**Figure 4:**
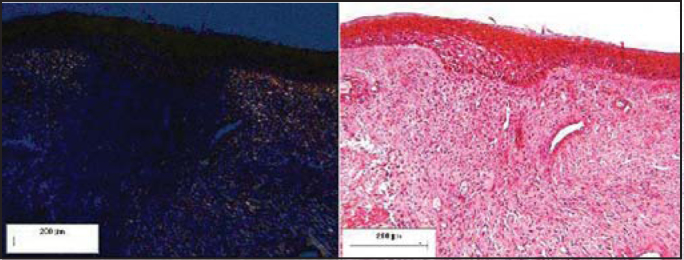
Pediculated flap placed on the submucous bed of the tested dog (postoperative day 10). Staining: Syrius Red under polarized light and HE.

**Figure 5:**
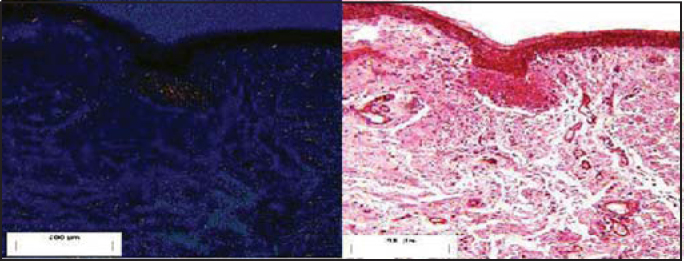
Pediculated flap placed on the submucous bed of the tested dog (postoperative day 30). Staining: Syrius Red under polarized light and HE.

Laminae were analyzed through optical microscope Olympus BX50 (Olympus Corporation, Ishikawa, Japan), and images were digitalized through the camera Nikon DXM 1200 (Nikon, Melville, New York) and transferred to the computer Pentium IV, using the application Image Pro-Plus version 4.5.1.22 for Windows Media Cybernetics.

After histological preparation, we had the slides stained with Syrius Red and HE of 3 studied vocal folds in 5 different postoperative periods, plus 15 control vocal folds. The values of total, type I and type III collagen and the number of cell nuclei in each tested group with 3 dogs corresponding to postoperative days 10, 30, 90, 180 and 360 were descriptively and analytically studied. Mean and standard deviation measures were used to describe the sample. To compare the studied groups with the control group, we used U test by Wilcoxon-Mann-Whitney to analyze small samples, defining α= 0.05 as the parameter for null hypothesis.

## RESULTS

On the slides corresponding to 10 postoperative day in the tested group, pediculated flap could be identified in the area corresponding to lamina propria superficial region. After this period, only the area of the pedicle could be identified on day 30 (3/3), day 90 (2/3), day 180 (2/3) and day 360 (2/3). Total collagen area measured in the 15 vocal folds in the tested group ranged from 688.58m^2^ to 4097.22μ^2^; standard deviation of 1155.31 and median of 1715.62 μ^2^; in the control group, the area ranged from 1002.21μ^2^ to 3395.33μ^2^, with standard deviation of 734.33 and median of 2054.87μ^2^. Type III collagen area in the 15 vocal folds of the studied group ranged from 58.05μ^2^ to 1479.38 μ^2^, standard deviation of 241.98 and median of 261.62m^2^; the area of the control group ranged from 58.27μ^2^ to 1028.38μ^2^, standard deviation of 248.48 and median of 475.94μ^2^.

Type I collagen area in the tested group ranged from 595.17μ^2^ to 3958.30μ^2^, the standard deviation was 1229.64 and median was 1113.28μ^2^; the area of the control group ranged from 286.23μ^2^ to 2695.80μ^2^, standard deviation was 1229.64 and median was 1491.46μ^2^.

The number of cell nuclei in the tested groups ranged from 21 to 79 nuclei, standard deviation of 17.51 and median 39. In the control group, the variation of nuclei was 8 to 89, standard deviation of 23.88 and median of 27.

**Graph 1:**
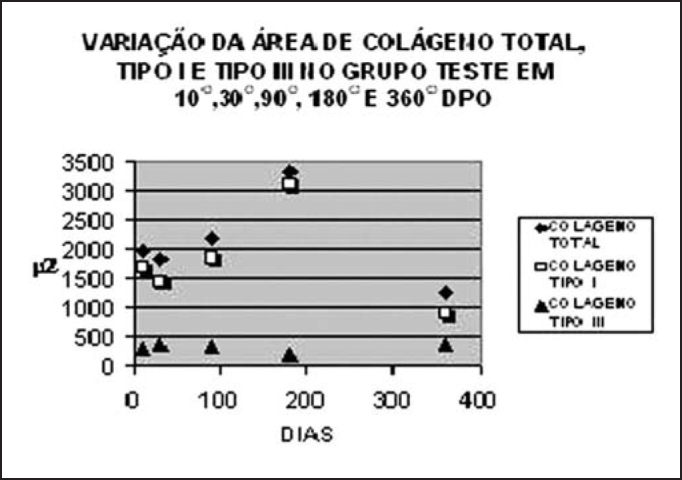
Variation of mean area of total, Type I and Type III collagen in postoperative period (n=3 for each period of observation).

The variation of total and type I collagen during the period of observation suggested a tendency of increase in tested groups on days 90 and 180 after the surgery, followed by drop on day 360 (Graph 1, Tables 1 and 2). On postoperative day 30, means of total and type I collagen in the studied group were below what we had for the control group (Tables 1 and 2). However, in the analyzed subgroups (n=3), concerning total and type I collagen, only those values related to day 180 presented statistically significant difference (p<0.05) compared to the control group (n=15) after application of Wilcoxon-Mann-Whitney U test.

**Figure 6:**
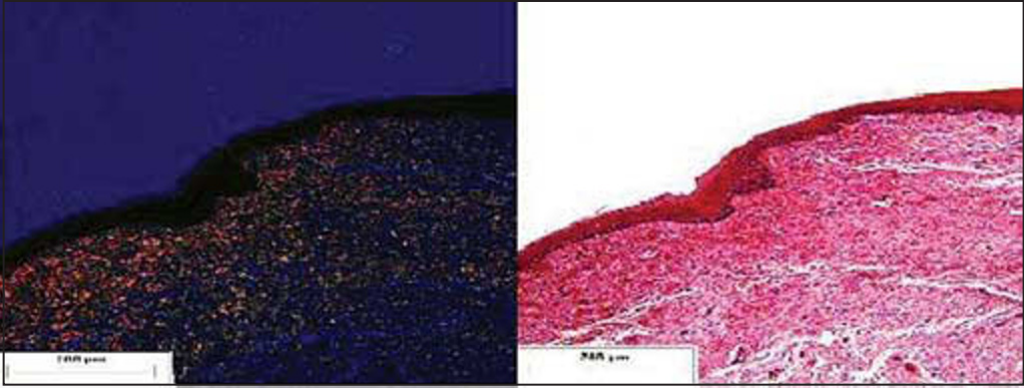
Pediculated flap placed on the submucous bed of the tested dog (postoperative month 12). Staining: Syrius Red under polarized light and HE.

**Table 1:** Total, type III and type I collagen and number of cell nuclei. Calculation of the mean of each postoperative period.

		Total	Collagen (μ^2^) Type III	Type I	Cell Nuclei
	DOG 1	3907,85	83,44	3824,41	44
10	DOG 2	688,58	93,4	595,18	79
DPO	DOG 3	1330,73	718,78	611,95	51
	MD 10 DAYS	1975,72	298,53	1677,18	58
	DOG 4	1741,01	58,05	1682,96	53
30	DOG 5	1348,96	478,29	870,67	24
DPO	DOG 6	2396,14	591,93	1804,21	36
	MD 30 DAYS	1828,7	376,09	1452,61	38
	DOG 7	1715,62	602,34	1113,29	64
90	DOG 8	1027,49	82,77	944,72	21
DPO	DOG 9	3794,55	294,4	3500,15	29
	MD 90 DAYS	2179,22	326,5	1852,72	38
	DOG 10	4097,22	138,92	3958,3	29
180	DOG 11	3303,84	136,69	3167,16	47
DPO	DOG	2590,43	335,9	2254,54	27
	MD 180 DAYS	3330,49	203,83	3126,66	34
	DOG 13	1080,18	261,63	818,55	21
360	DOG 14	1214,96	124,94	1090,02	39
DPO	DOG 15	1479,39	696,52	782,87	62
	MD 360 DAYS	1258,17	361,02	897,15	41

** MD= mean, DPO: postoperative day, μ^2^= squared micrometer

**Table 2:** Area of total, Type III and Type I collagen and number of cell nuclei in the control group. Calculation of mean and median.

	Total	Collagen (m2) Type III	Type I	Cell Nuclei
DOG 1	2187,31	58,28	2129,04	15
DOG 2	2151,52	660,05	1491,47	27
DOG 3	1407,8	394,73	1013,06	20
DOG 4	1932,06	368,22	1563,84	8
DOG 5	3395,34	699,54	2695,8	18
DOG 6	2532,83	657,59	1875,24	52
DOG 7	2054,88	843,72	1211,16	13
DOG 8	1002,21	463,19	539,03	15
DOG 9	1890,67	609,38	1281,29	69
DOG 10	2301,4	432,88	1868,53	28
DOG 11	2027,92	774,03	1253,89	35
DOG 12	2469,86	475,94	1993,92	63
DOG 13	828,28	363,75	464,53	43
DOG 14	834,32	248,09	286,24	23
DOG 15	2922,42	1028,39	1894,03	89
MEAN	1995,92	538,52	1437,4	34,5
MEDIAN	2054,87	475,94	1491,46	27

MD= mean, μ^2^= squared micrometer

Means of type III collagen area in the studied groups were always lower than those in the collagen groups (Graph 1) and reached levels below those of the control group (Tables 1 and 2) during the whole period of observation. Considering the postoperative period, only day 180 presented statistically significant reduced values (p<0.05).

There was no statistically significant correlation in the different periods of postoperative observation concerning number of nuclei on the lamina propria surface layer between the tested and control groups. There was only an initial increase on the average of number of nuclei in the studied group on day 10 PO (postoperative), followed by drop after this period (Tables 1 and 2).

## DISCUSSION

Authors such as Duke and Shaw considered that there is still no ideal implantable substance to apply on the lamina propria of vocal folds18., 19.. In the studies carried out by Mikus et al.^20^, we confirmed that there has been a very heterogeneous absorption of fat, causing variation between 2.3% and 70%, in addition to worsening of vocal quality within 3 to 6 months after the surgery. Duke et al.^18^ suggested the feasibility of fascia graft up to one year. However, postoperative results can be influenced by early extrusion or absorption. Courey^12^ suggested that the implantation of collagen on the lamina propria superficial layer has potential effects concerning local reduction of viscosity.

The flap developed by the present technique followed the vascular pathways parallel to the free margin. Analyses made by Bardach^21^ defined that for the feasibility of a skin flap it is required to have a flow of about 1 to 2ml per minute for 100cc of tissue. The proposed flap in our study required 0.004ml/minute. Therefore, this measure was not made.

The observation of the chronological distribution of total and type I collagen in the studied group suggested an increase in its levels on days 90 and 180 PO, but there was statistically significant difference only on day 180. Rosseau and Thibeault22., 23. studied the scarring process of lamina propria and noticed that pro-collagen was more present in the 2nd month postoperative, with stabilization of levels in the 6th month, the same period in which there were higher density collagen fibers manifested on the surgical wound.

After 180 PO day, there was reduction on the mean of total and type I collagen, with similar levels to the control group. Few experimental studies of the larynx are extended for more than 6 months, but further studies are required to reach better conclusions about longer periods of observation.

The comparison of measurements obtained in the group that received the pediculated flap with the two studied groups from Cervantes et al.^24^ is suggestive of the fact that the present study had lower levels of total collagen on day 30 PO. This piece of data may be comparable with less local fibrosis, but, owing to the reduced number of dogs tested in the present study (n=3), nothing could be confirmed.

The mean area of type III collagen in the tested group was below that of the control group, but there was statistically significant difference only on day 180 PO.

Findings related to analysis of type III collagen differ partially from other studies^22^,^23^, which noted that pro-collagen (immature form of collagen) was maintained at higher levels than those measured in the control group up to month 2, in addition to the fact that in month 6 there was simultaneous decrease of pro-collagen and increase of more dense collagen.

The analysis of extracellular matrix behavior after application of surgical technique of pediculated flap revealed that there were significant changes concerning the increase in the area of more dense collagen (type I), associated with reduction in the area of immature or less dense collagen (type III) on day 180 PO. It may suggest similarities with postoperative findings of other experimental studies in the larynx, except for persistent low levels of Type III collagen during the period of observation.

More comprehensive studies are required, comprising a control group in which a simple incision is made and with the use of specific antibodies to identify different subtypes of collagen fibers, elastic fibers, fibronectin, hyaluronic acid, other aminoglycan, proteoglycan, lipids and carbohydrates are necessary to better understand the impact of pediculated flaps on the lamina propria superficial layer.

The means of number of nuclei in the tested groups reached high levels on 10 day PO, progressing for the decrease after day 30. These may be data compatible with the scarring process on the transition of proliferative phase to the remodeling phase^25^, however, we did not find statistically significant results in any of the observed periods.

Postoperative observations made under contact laryngoscope did not detect the onset of compatible lesions with vocal cysts, as well as significant affections in vascularity of the site where the flap was positioned. It is worth reporting that the present study was performed in dogs and their lamina propria has more percentage collagen than human lamina propria, especially on the superficial layer^17^. The analysis of collagen, therefore, can be more accurate for the regeneration in dogs than in men.

The present study was unheard-of concerning the prolonged time of postoperative observation in dogs (10 days to 1 year) and concerning the introduction of a new surgical technique. Some issues could not be completely answered with the results obtained, but they also gave rise to new perspectives, looking for checking complete and complex responses.

## CONCLUSIONS

The study showed that in the group of dogs in which the technique of vocal fold pediculated flap was applied there was tendency to increase the levels of total and type I collagen on the lamina propria surface layer on days 90 and 180 PO, followed by drop on day 360; however, statistically significant difference was found only on day 180 PO.

The mean of type III collagen in the studied group was below that of the control group in all periods of observation; however, there was statistically significant difference on day 180 PO.

The number of cell nuclei in the tested group was higher on day 10 PO, followed by reduction on levels after day 30, but we did not find statistically significant results in any of the observed periods.
